# Animal Model Contributions to Primary Congenital Glaucoma

**DOI:** 10.1155/2022/6955461

**Published:** 2022-05-26

**Authors:** Qiongrong Xia, Dingding Zhang, Yue Zhuang, Yuqian Dai, Haiping Jia, Qiu Du, Taishen Wen, Yuanyuan Jiang

**Affiliations:** ^1^Primary Congenital Glaucoma Research Group, College of Medical Technology, Chengdu University of Traditional Chinese Medicine, Chengdu, China; ^2^Sichuan Provincial Key Laboratory for Disease Gene Study, Sichuan Provincial People's Hospital, University of Electronic Science and Technology of China, Chengdu, China; ^3^Department of Rheumatology and Immunology, Sichuan Provincial People's Hospital, University of Electronic Science and Technology of China, Chengdu, China; ^4^School of Medicine, St. George's University, New York, USA; ^5^Department of Immunology, North Sichuan Medical College, Nanchong, China

## Abstract

Primary congenital glaucoma (PCG) is an ocular disease characterized by congenital anterior segmental maldevelopment with progressive optic nerve degeneration. Certain genes, such as cytochrome P450 family 1 subfamily B member 1 and latent TGF-*β*-binding protein 2, are involved in the pathogenesis of PCG, but the exact pathogenic mechanism has not yet been fully elucidated. There is an urgent need to determine the etiology and pathophysiology of PCG and develop new therapeutic methods to stop disease progression. Animal models can simulate PCG and are essential to study the pathogenesis and treatment of PCG. Various animal species have been used in the study of PCG, including rabbits, rats, mice, cats, zebrafish, and quails. These models are formed spontaneously or by combining with genetic engineering technology. The focus of the present study is to review the characteristics and potential applications of animal models in PCG and provide new approaches to understand the mechanism and develop new treatment strategies for patients with PCG.

## 1. Introduction

**Figure 1:**
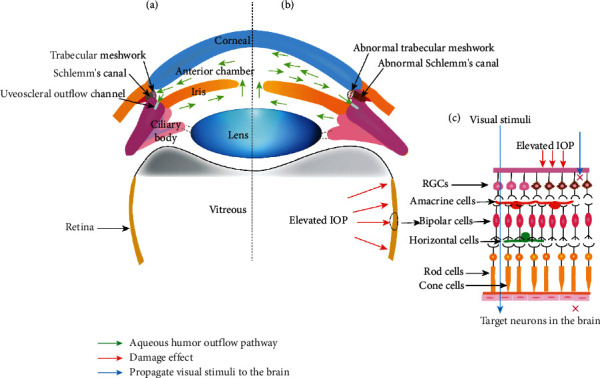
Schematic diagram of the circulation of aqueous humor. (a) Normal aqueous humor circulation. The aqueous humor is drained mainly through the conventional outflow channel (mainly composed of trabecular meshwork and Schlemm's canal) and the uveoscleral outflow channel. (b) Abnormal anterior chamber angle. In primary congenital glaucoma patients, dysplastic trabecular meshwork and Schlemm's canal impaired aqueous humor outflow. Obstruction of the aqueous humor outflow leads to a continuous increase in intraocular pressure. (c) Elevated intraocular pressure affects retinal nerve function. Persistently elevated intraocular pressure leads to optic nerve damage, which eventually causes the death of retinal ganglion cells and disrupts the propagation of visual stimuli to target neurons in the brain. IOP: intraocular pressure; RGCs: retinal ganglion cells.

Primary congenital glaucoma (PCG) is one of the leading causes of childhood blindness and is a characteristic of incomplete development of the trabecular meshwork (TM) and anterior chamber angle with optic nerve degeneration [1, 2]. Worldwide, the overall incidence of PCG is 1–80 patients per 100,000 live births [3]. In the highly consanguineous subpopulation of Slovakia, the incidence of 1 : 1250 individuals has been reported [4]. The incidence of PCG in offspring may be 5 to 10 times higher if there is consanguinity in the parents [2]. PCG cases are unilateral or bilateral, showing variable penetrance (40–100%) and various severities [5]. Elevation in intraocular pressure (IOP) is a common risk factor that threatens the survival of retinal ganglion cells (RGCs) and favors disease progression. The mechanism to maintain IOP is a normal circulation of aqueous humor (AH) through the drainage pathway (Figure 1).

PCG is an inherited disorder with strong monogenetic influence [2]. Four distinct genetic loci related to PCG have been identified by genetic linkage analysis; that is, GLC3A in the chromosome region 2q21-22, GLC3B in 1p36.2-36.1, GLC3C in 14q24.3-31.1, and GLC3D in 14q24 [6–9]. GLC3A was the first locus to be found in 17 Turkish families [6]. The cytochrome P450 family 1 subfamily B member 1 (*CYP1B1*) is located in GLC3A. More than 150 variants of *CYP1B1* have been found in PCG cases worldwide, accounting for 87% of cases in inbred populations [10, 11]. GLC3B was the second locus, identified in 8 families, 17 of whom had PCG [7]. Cornea-derived transcript 6 (*CDT6*) is a probable candidate gene at the GLC3B locus and may regulate the deposition of specific extracellular matrix (ECM) components in glaucoma [12–14]. In addition, GLC3C and GLC3D were reported to be associated with PCG. Among them, the GLC3C locus may be linked to latent transforming growth factor-beta binding protein 2 (*LTBP2*) [8, 15]. In recent years, more genes have been implicated in PCG, such as forkhead box C1 (*FOXC1*), angiopoietin 1(*ANGPT1*), TEK receptor tyrosine kinase (*TEK*), G-patch domain containing 3 (*GPATCH3*), and guanylate cyclase activator 1C (*GUCA1C*) [16–19]. However, the pathogenesis of PCG needs to be further explored.

Various animal models of glaucoma have been described in other review articles [20, 21]. At present, animal models used in PCG include rabbits [22], rats [23], mice [24], cats [25], zebrafish [26], and quails [27]. The study of these models provides valuable information on the pathophysiology of PCG, as it is associated with changes in the angle of the anterior chamber, the optic nerve, and the retina, although the mechanisms that caused these changes are unclear. The purpose of the present study is to review the pathological features of animal models and the possible genetic mechanisms associated with PCG, which would provide references for future modeling and pave the way for the elucidation of the pathogenic mechanism of PCG.

## 2. Genes Related to PCG

Although exact causative genes and pathways have yet to be confirmed, several related genes, including *CYP1B1*, seem to be involved in the development of the anterior segment of the eye, and they may participate in the progression of PCG (see Table 1).

### 2.1. CYP1B1

The biallelic variants of *CYP1B1* represented approximately 64.8% of PCG cases in the Middle East and 54.4% of PCG cases in the Maghreb [41]. Similarly, the rate of *CYP1B1*-mutated alleles was 34.7% in Europe, 21.3% in Asia, and 14.9% in the United States [41]. There exists a strong clinical genotype/phenotype correlation with some variants causing PCG with histopathological features, while A330F caused by c.988G>T&c.989C>T and R390H caused by c.1169G>A are associated with the severe glaucomatous phenotype [42].

Gene product loss-of-function (LoF) caused by *CYP1B1* mutations has been reported to be involved in the development of TM of PCG. The CYP1B1 enzyme metabolizes a signaling molecule required in the development of ocular tissues (possibly endogenous steroid metabolism) and eliminates toxic metabolites [10, 43, 44]. Meanwhile, 17*β* estradiol, a *CYP1B1* metabolite, is involved in the cAMP/protein kinase A pathway together with *MYOC* to affect TM formation [45–47]. However, the endogenous target in vivo is still unknown. In zebrafish, *CYP1B1* was expressed in the dorsal and ventral retina in an ocular fissure, overlapped with retinoic acid (RA) synthesis enzymes (RALDH2 and RALDH3) [28, 48]. *CYP1B1* is a component that mediates the conversion of vitamin A (Vit A) into the retina and then into RA by the dehydrogenase-independent pathway [48]. The overexpression of *CYP1B1* inhibited ocular fissure closure, causing colobomatous defects [28]. By comparison, *CYP1B1* knockdown led to the premature closure of inferior optic fissure and changes neural crest (NC) later migration via the RA-independent pathway [28]. Furthermore, the formation of iris stroma derived from the crest and the recovery of retinal development were delayed in the larval stage in *cyp1b1*-knockdown zebrafish [49]. Null *CYP1B1* activity altered the expression of ECM, lipid metabolism genes, and inflammation [50].

Other findings reported that *CYP1B1* is an important regulator of redox homeostasis, which is related to increased oxidative stress in trabecular meshwork cells (TMCs) and retinal vascular cells in PCG [51]. And the increased oxidative stress alters the production of ECM protein periostin (Postn) that influences the cellular integrity and function of TM [51, 52]. Postn is an ECM protein that interacts with collagen fibers and is involved in TM's function and morphology [53]. Moreover, *CYP1B1* metabolite, arachidonic acid, regulates the transparency of the cornea and the function of the Na^+^-K^+^ ATPase in corneal microsomes [54, 55]. Thus, the later migration of NC mediated by *CYP1B1* in proper spatial-temporal scope is essential for ocular tissues to form. Also, *CYP1B1* contributes to the maintenance of the homeostasis of structure-function of TM tissue through regulating the expression of Postn [29].

### 2.2. LTBP2

Ali et al. reported that p.R299X in *LTBP2* was the main variant of the founder of PCG in the Gypsy population, which accounted for more than 50% of *CYP1B1*-negative cases and ∼40% of all PCG cases [15]. *LTBP2* is identified as a PCG-associated causative gene and is expressed in the TM and ciliary body, particularly in the ECM protein of the ciliary body [15, 31]. The homonymous protein encoded by *LTBP2* is an ECM protein and is thought to be a member of latent *TGF-β* complex and a structural component of microfibrils. *LTBP2* plays an important role in cell adhesion and supporting ciliary muscle tone, binding to fibrillin-1 through the carboxyl-terminal (C-terminal) and fibulin-5 through the amino-terminal (N-terminal) [56, 57]. Ali et al. demonstrated that *LTBP2* mutations increased the elasticity of ciliary body tissue and altered the structural support of the context tissues [15].

**Figure 2:**
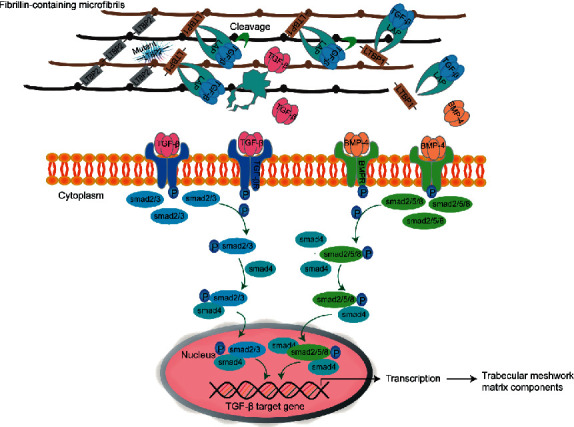
*LTBP2* indirectly mediates the *TGF-β/BMP* signal pathway to regulate elastogenesis or anchors to components of the matrix of the trabecular meshwork. Mutant *LTBP2* loses competition with *LTBP1* for fibrillin-containing microfibrils, which increases the concentration of activated *TGF-β* and upregulates *TGF-β* signal to activate target gene. *BMP* signal negatively regulates the *TGF-β* signal to downregulate *TGF-β* gene expression. LTBP2: latent transforming growth factor-beta binding protein 2; TGF-*β*: transforming growth factor-beta; BMP: bone morphogenetic protein; LAP: latency-related protein; TGF-*β*R: transforming growth factor-beta receptor; BMPR: bone morphogenetic protein receptor.

Furthermore, *LTBP2* may indirectly negatively regulate the concentration of the large latent *TGF-β* complex on microfibrils [56]. A hypothesis is that *LTBP2* competes with *LTBP1* to bind to the same binding site in fibrillin-containing microfibrils, leading to the release of *LTBP1* from microfibrils [56, 58]. By strong noncovalent bonds, *TGF-β* binding to latency-related protein (LAP) with *LTBP1* forms the large latent complex (LLC) [58]. When LAP is integrated by integrating the cell surface, *LTBP1* promotes the activation of *TGF-β* via fixing to the ECM to produce traction [58]. This traction leads to deformation of the LAP to release active growth factor [58]. Active *TGF-β* is released and *BMP* binds to the domain of their receptors, and phosphorylation of the serine/threonine kinase of receptors results in activation of the SAMD pathway to transmit the signal (Figure 2) [58, 59]. Suri et al. confirmed that *LTBP2* had a putative effect on TGF-*β* signaling and the ECM of TM [32]. *LTBP2* knockdown increased samd2 phosphorylation and decreased phosphorylation of samd1/5/8, suggesting *LTBP2* knockdown promotes *TGF-β* signaling and inhibits the bone morphogenetic protein (*BMP*) signaling pathway [32]. It suggested *LTBP2* affecting the balance of *TGF-β/BMP* signaling is essential to maintain the steady structure of ECM in TM and mediates the apoptosis of TMCs [32, 60].

### 2.3. FOXC1

It has been shown that *FOXC1* variants are a common cause of Swiss childhood glaucoma [61]. Medina-Trillo et al. reported five rare heterozygous *FOXC1* variants ((rs77888940, c.-429C>G; rs730882054, c.1134_144delCGGCGGCGCGG; rs35717904, c.^*∗*^734A>T; rs185790394, c.-244C>T; and rs79691946, c.^*∗*^454C>T) in 10 patients without a family history of glaucoma, and this indicated that *FOXC1* variants may contribute to the formation of goniodysgenesis in PCG [62]. The expression of FOXC1 is in periocular mesenchyme (POM) derived from the NC and appears to be related to the development of iridocorneal angle. Seo et al. showed that NC-*FOXC1*^*−*/*−*^ reduced the expression of paired like homeodomain 2 (*PITX2*) and downstream effector dickkopf WNT signaling pathway inhibitor 2 (*DKK2*), suggesting that *FOXC1* as a regulator in *WNT* signaling regulates and maintains ocular development [33]. In addition to that the expression of *PITX2* and *FOXC1* depends on RA signaling in the POM [34, 63, 64]. *TGF-β* directly regulates *FOXC1*- and *PITX2*-positive cells to differentiate into corneal endothelial and stromal cells postmigration [35].

### 2.4. *ANGPT1* and *TEK*

*ANGPT1/TEK* plays an essential role in vascular development and is also implicated in the development of Schlemm's canal (SC). Dysplasia of SC and TM in AH drainage was frequently observed in PCG patients with elevated IOP [65]. This indicates that the *ANGPT1/TEK* pathway is involved in the development of AH drainage structures in PCG. Souma et al. identified 10 rare variants of *TEK* in 189 pedigrees with PCG, and each variant resulted in haploinsufficiency due to the LoF protein [17]. Similarly, Thomson et al. reported that 3 variants (p.Q236^*∗*^, p.R494^*∗*^, and p.K249R) of *ANGPT1* were identified in the cohort of 284 PCG patients, and *ANGPT1* mutations reduced the ability to activate *TEK* signaling [66].

*TEK*, a receptor tyrosine kinase, regulates vascular homeostasis by its autophosphorylation and transphosphorylation, which is highly expressed in the endothelium of SC [17, 67, 68]. *ANGPT1*, which is a primary ligand expressed in pericytes and other vascular supporting cells, plays a significant role in proangiogenic and vascular stabilization by activating *TEK* [66]. Then, vacuoles formed from the endothelium of SC, which subsequently resulted in SC endothelial thinning and formation of pores to induce the normal flow of AH [69,70]. It has been reported that disruption in the *ANGPT1/TEK* pathway possibly caused PCG-like phenotypes, such as elevated IOP, RGC degeneration, and buphthalmos [71]. Several studies have shown that the *ANGPT1/TEK* pathway supports and maintains the stability of the function of SC and TM, which is one of the pathological mechanisms of PCG [17, 66].

### 2.5. GPATCH3

Ferre-Fernández et al. identified two heterozygous variants (c.701A>G, p.Asn234Ser and c.1424G>A, p.Gly475Glu) of *GPATCH3* in a PCG family [18]. The disease phenotype in the PCG family caused by *GPATCH3* variants was autosomal recessive inheritance [18]. It was predicted that c.701A>G, p.Asn234Ser and c.1424G>A, p.Gly475Glu affected highly conserved amino acid residues and were disruptive [18]. The variants of *GPATCH3* showed that nuclear localization was similar to *FOXC2*. Embryonically, the pattern of *GPATCH3* expressed is similar to that of *PITX2* and overlaps with *FOXC1* presented in the POM [18]. *GPATCH3* encoded a protein with the G-patch domain, which was present in both RNA-binding and DNA-binding and participated in protein-nucleic acid interactions [72]. It is assumed that *GPATCH3* regulates iridocorneal angle development by activating the GPATCH3 protein that activates the promoter of the C-X-C motif chemokine receptor4 (CXCR4) to regulate DNA sequences or indirectly mediates the mechanisms involved in PCG [18]. Compared with the wild-type protein (WT), the affected protein increased ∼17% of the transactivation activity of CXCR4, suggesting that changes in amino acids caused hypermorphic variants and led to functional alterations of the affected protein [18]. *CXCR4* is linked to NC migration through encoding chemokine receptors [38]. This provides evidence that the function of *GPATCH3* may interact with *PITX2* and *FOXC1* and that *GPATCH3* is implicated in PCG [18].

### 2.6. GUCA1C

Morales-Cámara et al. identified one homozygous variant of *GUCA1C* (c.52G>T; p. Glu18Ter) in two affected siblings with PCG by an autosomal recessive pattern [19]. It is predicted that c.52G>T in *GUCA1C* resulted in complete LoF of the gene product [19]. The GUCA1C-encoded guanylate cyclase 3 activating protein (GCAP3) belongs to the member of the guanylate cyclase-activating protein family, which is related to phototransduction and regulates IOP [19, 39, 73]. GCAP3 was expressed in the iris tissues, corneal tissues, retina, photoreceptors, and RGCs, suggesting that GCAP3 regulated the homeostasis of IOP and that *GCAP3* LoF may contribute to congenital glaucoma [19]. Furthermore, *GUCA1C* reduces IOP by regulating the volume of SC and TMCs in a cGMP-dependent manner to increase the outflow of AH [19, 39]. However, to prove whether the *GUCA1C* variant causes PCG, more experimental data are required [40].

## 3. Treatment Methods for PCG

Currently, clinical treatments for PCG include surgery and drug-assisted treatment. Carbonic anhydrase inhibitors (CAIs) are aimed at inhibiting the secretion of bicarbonate ions and reducing the flow of liquid to reduce the production of AH in the ciliary body [74, 75]. Compared with *β*-receptor blockers, CAIs effectively reduce IOP by ∼25% with fewer side effects but will cause anorexia, thirst, fatigue, renal acidosis, and growth retardation in the long term [76, 77]. Furthermore, certain medications, for example, latanoprost, the prostaglandin F2*α* (FP) receptor agonist, increase the amount of AH in the uveoscleral outflow pathway [78, 79]. Uva et al. pointed out that the mean reduction in IOP controlled by latanoprost in children was 35.6% and lasted 18 ± 22 months, but adverse events included conjunctival hyperemia, increased pigmentation of the iris, changes in eyelashes, and irritation of the upper airways [80].

So far, almost all cases of PCG require initial surgery because medications are not always effective or feasible and are poorly tolerated long term in pediatric patients [2, 81]. Goniotomy is the first choice in PCG because the conjunctiva is preserved and therefore does not jeopardize the success of procedures in the future [82]. The advantage of trabeculotomy is that it possibly is used in eyes with corneal opacity and may have a higher success rate than goniotomy [81, 83]. Trabeculectomy is performed after other procedures failed, with the overall goal of allowing AH to outflow from the anterior chamber into the sub-Tenon space [84, 85]. The success rate of trabeculectomy varies from 55.3% to 92.3% and has a high rate of complications, such as endophthalmitis and other herpes-related infections (6.7%) [81]. More procedures also are performed to increase AH outflow for PCG, for example, filtration surgery, cyclodestructive procedure, and combined trabeculotomy and trabeculectomy [86–88]. The advantages and success rates of these procedures are varied, and their application should be weighed against the effectiveness of each method and the complexity of the disease [81]. Preoperative and postoperative medications are used to help control IOP and prevent refractive error, amblyopia, and other secondary complications [86, 89, 90]. Importantly, long-term follow-up and lifelong control of IOP are necessary.

The independent anatomical structure of the eyes, easily transduced cell population, and the unique anatomical barrier and physiological environment of the eyes constitute suitable conditions for gene therapy [91]. The preferred target tissue to reduce IOP is TM while for neuroprotection is RGCs [92]. The progress made in gene therapy brings hope to the treatment of ophthalmic diseases, especially in PCG. Recently, advances and progress made in gene therapy of glaucoma bring hope to the treatment of PCG [93]. Connective tissue growth factor was reported to be inhibited by the AAV-CRISPR-Cas9 system in rabbit glaucoma, which improved the outcomes of glaucoma filtration surgery [94]. Yang et al. reported that delivery of exogenous *p27* to the rabbit model would inhibit the proliferation of Tenon's capsule fibroblasts at glaucoma surgery sites and the formation of scars, thus improving the efficacy of surgery [95]. Silencing the P2Y_2_ receptor by siRNAs in rabbits reduced IOP by 67%, and the treatment effect lasted for 5 days [96]. Similarly, silencing of *GAS5* relieved symptoms of rat glaucoma by rescuing RGC apoptosis [97]. Jiang et al. pointed out that treatment of glaucoma in a rat model with superoxide dismutase (SOD2) mediated by recombinant adeno-associated virus (AAV) may protect RGCs from chronic IOP elevation damage [98]. Zhou et al. reported that AAV-mediated *Map2k1* gene transfer into the RGCs of rats significantly increased neurons' survival from 680 ± 86 RGCs/mm^2^ to 1366 ± 70 RGCs/mm^2^ [99]. Similarly, Luna et al. indicated that delivery of miR-146a provided a long-term reduction of IOP without inflammation in a rat model [100]. These findings suggest that gene therapy may be promising for patients with PCG. However, gene therapy for PCG has a long way to go, and identifying the causative gene is a prerequisite for it.

## 4. Animal Models Related to PCG

Currently, animal models are divided into two categories, the spontaneous model (see Table 2) and the genetic model (see Table 3), which also elucidate the clinical phenotype and pathological mechanisms of PCG from different perspectives.

### 4.1. Spontaneous Models

#### 4.1.1. Rabbit Model

The rabbit model with PCG is mainly spontaneously inherited. Congenital abnormalities in anterior chamber development in rabbits with PCG were similar to those in humans. Abnormalities included corneal epithelial (sometimes stromal) edema, larger corneal diameter, hyperemia of the ciliary body, compression or loss of the iris pillars, increased cell cornification, and hypoplasia or posterior displacement of the aqueous plexus [101, 102]. Additional findings included loss of association of trabecular endothelial cells to cells, disorganization of trabecular lamellae, compression or dilation of intertrabecular spaces, and a decrease in the number of collagen fibers [102]. Others reported that TM was replaced by abundant ECM, unidentified round cells below the aqueous plexus, and basement membrane-like material [114].

In the rabbit model, except for the typical manifests mentioned, variable elevation of IOP (up to 48 mmHg), retina nerve degeneration, and outflow facility defect were observed [101, 120]. An important finding was that there was no marked blockage of axonal transport in rabbits when IOP was elevated acutely because rabbits lack a true lamina cribrosa (LC) [120]. This was consistent with the hypothesis that elevated IOP mechanically damages the optic nerve. PCG in rabbits is likely to be inherited through an autosomal recessive pattern with incomplete phenotypes and penetrance [121]. Gene mutation in rabbits inactivated genes of TM development and eventually led to maldevelopment of TM tissue [22, 101]. In addition, a lack of vitamin A may be associated with the severity of PCG, as it participates in the stability of the cell's membrane structure and maintenance of the optic nerve [101]. Furthermore, in rabbit models, abnormal expression of AH proteins in PCG led to a decrease in the number of RGCs, thickening of Descemet's membrane and anterior lens capsule [103].

However, interest in the rabbit model gradually decreased as genetic models of PCG became available. Ishida et al. performed trabeculectomy and Ex-PRESS filtering to treat PCG in rabbits, respectively, and demonstrated that the overall effect of trabeculectomy on blood pressure reduction was relatively stable [122]. The inflammation of Ex-PRESS filtering was milder than that of trabeculectomy. Furthermore, nanostructured glaucoma drainage implants have also been successful in rabbit models, showing good tolerance, minimal leakage of implants, good compatibility with cells and tissues, and IOP reduction by 33–44% [123]. These studies suggest that the rabbit model is ideal for simulating surgical treatment.

The rabbit model of PCG should avoid the interference of conjunctivitis, which is a common disease in rabbits caused by infectious and/or noninfectious diseases during the experiment [124]. And the intermittent elevation of IOP may lead to unstable experimental results [114]. Furthermore, distinctions in the anatomical structure of TM and the drainage route of AH make it difficult to establish an anterior chamber angle association between rabbits and humans [125]. The SC drainage function replaced by the aqueous plexus in rabbit models restricts the ability to study the genetic mechanism of PCG [103]. In addition, genetic testing in rabbits is restricted due to the limited rabbit genomic resource available for testing [103]. The *CYP1B1* mutation in rabbits could not be identified [103].

#### 4.1.2. Rat Model

In spontaneous rat PCG models, like rabbits, it showed symptoms similar to humans [23, 105, 106]. Additionally, the rat model showed unilateral or bilateral enlarged globes with the IOP ranging from 29 to 42.5 mmHg [105, 107]. After 18 months, the rat model showed that the remaining RGC was 92 ± 26 RGC/mm^2^, while the WT group was 1887 ± 117 RGC/mm^2^ [107]. Furthermore, the condition worsened with age, showed mitochondrial dysfunction, lack of nutritional proteins, accumulation of toxic substances, and oxidative stress [107]. Others reported upregulation of *c-Myc* in the RGCs and neurons of the inner nuclear layer promoted cell differentiation and induced cell apoptosis [106]. Elevated levels of heat shock protein 27 and VEGF in RGCs exerted a protective effect on RGCs, while the upregulation of glial fibrillary acidic protein, growth-associated protein 43, and endothelin-1 in glial cells promoted the apoptosis of RGCs [106, 107]. Additional findings included large anomalous tissue deposition accumulated in the suprachoroidal space, and the uveoscleral outflow pathway was severely impaired [108]. These rat models used in PCG offer valuable information on both RGC loss and uveoscleral outflow.

Although the rat is a common tool for studying disease in the laboratory, some questions need to be considered. First, the TM of rats is less intensive than that of humans and cannot generate enough flow resistance because it is a few layers thick [109, 126]. Second, differences in LC and vascular supply to the optic nerve head (ONH) between rats and humans need to be taken into account compared to the human situation [109]. In addition, rodents, such as rats and mice, do not have a fovea, their LC is faintly present and also both choroid and pial vessels possibly exist [104, 109]. Hence, the rat is not suitable to study LC-specific function.

#### 4.1.3. Cat Model

Cats with PCG showed IOP > 30 mmHg (normal IOP: 20 ± 5 mmHg), and those with nonspecific conjunctivitis were likely to suffer from glaucoma [25]. The red eye is an early sign of PCG in cats and indicates the optimal time for treatment [25]. In the spontaneous cat model, arrested vascular development, posterior displacement of intrascleral vessels, and loss of the RGCs resembled human PCG [25, 110, 111, 127]. The IOP in cats continued to increase until at least 6 months of age [127]. Elevated IOP ranged from 30 to 40 mmHg with intermittent IOP peaks of 50 to 70 mmHg [111]. The mean average retinal nerve fiber layer (RNFL) thickness of cats with PCG ranged from 8 to 20 *μ*m (normal RNFL is 40 *μ*m) [110].

Kuehn et al. reported the first case of hereditary glaucoma with complete penetrance in a domestic cat, which was caused by *LTBP2* mutation [112]. The sequence homology between cat and human orthologs reaches 87% over 100% length [112]. The *LTBP2* mutation in the cat model was shown to be autosomal recessive inheritance, and the postnatal development of the AH drainage system of the anterior segment was arrested [112]. Except for elevated IOP, cats were reported to have disrupted microstructural integrity [128]. The clinical appearance in cats with PCG included iris hypoplasia and ectopia lentis [112]. An open, slightly narrowed iridocorneal angle and mild maldevelopment of the pectinate ligament were observed by goniophotography [112]. The ONH cupping and degeneration in cats with advanced PCG were observed by fundus photographs [112]. Compared ∼83398 optic nerve axons in normal cats, PCG-affected cats had a moderate loss of axons, which remained ∼30365 optic nerve axons [112]. The AH outflow pathway composed of the uveal, corneoscleral TM, and angular aqueous plexus appeared collapsed [112]. Genetic analysis of the B3 chromosome *LTBP2* in domestic cats showed a 4 bp insertion and deletion in exon 8, resulting in a frame shift mutation that produces a truncated protein; the protein containing an abnormal C-terminal may accumulate in cells or become degraded and participate in the development of PCG [112].

However, in cats, there is no structure similar to human SC, and the uveoscleral route accounts for less than 3% of the AH outflow [129]. PCG also progresses slowly and degeneration of the ciliary body may cause inaccurate IOP measurements in cats [113]. Furthermore, creating cat disease models for PCG research is complicated by the need to remove various mixed phenotypes to guarantee a mono-disease reproductive population [111, 130].

#### 4.1.4. Quail Model

The pattern of inheritance in albino mutant quails is sex-linked recessive (*al* mutant) [27]. Albino mutant quails exhibited clinical features similar to other spontaneous glaucoma animals, such as enlarged eyes, RGC degeneration, and optic disc cupping [27]. The mean IOP in the *al* mutant quail was significantly higher than that in the normal quail at six months of age (25.2 ± 2.2 vs. 17.3 ± 1.7 mmHg), and some of them showed markedly elevated IOP that was over 35 mmHg at one year [27]. The opened iridocorneal angle, the shallowed anterior chamber, and the flat corneal curvature were observed in *al* mutant quails at 3 months [27]. In addition, in the ganglion cell layer, 11%–55% of the total number of RGCs decreased, resulting in a reduction of the iso-density map in the retina [115]. At the advanced stage, almost all RGCs had disappeared and the eye of the quail model showed anterior synechia [27, 115].

Thus, the quail model seems suitable for studying spontaneous glaucoma because of easy maintenance and handling in the laboratory [27]. However, the small size of the quail cornea makes IOP measurement a challenge [114, 115]. Furthermore, the *al* mutant has not been described in detail [114].

### 4.2. Genetic Models

No further reports have been published describing the clinical, pathological, and genetic characteristics of PCG in spontaneous models. Genetic models have validated some genes with altered expression and potential pathogenic signaling pathways in human PCG.

#### 4.2.1. Mouse Model

Mice are becoming the most common experimental animals in the laboratory because of their advantages, such as rapid reproduction, easy breeding, the highly conserved genome between mice and humans, and the availability of genetic manipulation [131]. The morphology of the iridocorneal angle and the development sequence of ocular structures in mice are similar to those of humans [109].

*(1) Cyp1b1-Deficient Model*. The *Cyp1b1* sequence homology between mice and human orthologs reaches 83% over 100% length (https://blast.ncbi.nlm.nih.gov/). A *Cyp1b1*-deficient mouse model has been developed to simulate PCG to further understand the molecular mechanisms. *Cyp1b1*^−/−^ mice also showed a genotype/phenotype correlation that was similar to that in PCG patients [30]. During the early stages of development of mice, spatiotemporal expression patterns showed that *Cyp1b1* was involved in the establishment and (or) maintenance of polarity along the axis of embryonic development [132]. The expression of *Cyp1b1* was in the retina, in the tissues around the optic nerve, and the developing and mature ciliary body of the mice eyes [24].

In *Cyp1b1-*deficient mice (*Cyp1b1*^−/−^), both the elevation of IOP and the ultrastructural abnormalities of ECM in TM were similar to those of human PCG [29, 30]. The mean IOP in *Cyp1b1*^−/−^ mice was 11.45 ± 0.20 mmHg, which was higher than that in *Cyp1b1*^+/+^ mice (10.14 ± 0.07 mmHg). At 3 weeks, *Cyp1b1*^−/−^ mice showed obvious disruption of the anterior and posterior TM, which were characterized by multifocal atrophy of the trabecular beams with the expansion of the intertrabecular spaces [30]. Marked fragmentation and irregular distribution of collagen fibers on the collagenous layer of the trabeculae were observed [30]. TMCs showed irregular cytoplasmic processes, a lack of attachment to the basement membrane, cytoplasmic vacuolation, and accumulation of organelle debris within the cytoplasm [30]. With increasing age, *Cyp1b1*^−/−^ mice exhibited progressively severely disarranged trabecular collagen. At 8 months, *Cyp1b1*^−/−^ mice exhibited complete collapse of the TM and formed an irregular atrophic trabecular beam across the anterior and posterior TM [30]. In addition, the relative amount of TM collagen was less and progressively decreased with age in *Cyp1b1*^−/−^ mice [30]. Zhao et al. also found severe morphological disruption in collagen fibers and increased intracellular oxidative stress in *Cyp1b1*^−/−^ TM tissues [29]. In response to stress, *Cyp1b1*^−/−^ TMC trended to apoptosis and the secretion of Postn was decreased [29].

Falero-Perez et al. determined the role of *Cyp1b1*^−/−^ in retinal endothelial cells (EC), pericytes (PCs), or astrocytes (ACs) on retinal neovascularization by developing cell-specific targeted *Cyp1b1*-transgenic mice [133]. During oxygen-induced ischemic retinopathy (OIR), attenuation of the pathological retinal neovascularization of PC-*Cyp1b1*^−/−^ mice was similar to the phenotype previously observed in *Cyp1b1*^−/−^ mice worldwide [133]. Also, *Cyp1b1*-transgenic mice exhibited destruction and loss of ECM and the degenerative cellular alterations in TM tissues [133]. This indicated that the expression of *CYP1B1* in perivascular supporting cells and EC affected the development of ischemia-mediated retinal neovascularization and maintains the integrity of TM by regulating cellular redox homeostasis. However, this work did not report the abnormal IOP in these mice.

So far, whether *Cyp1b1*^−/−^ causes IOP abnormalities and glaucomatous phenotypes in mouse models is controversial. Amirmokhtari et al. reported that IOP in *Cyp1b1*^−/−^ mice was within the physiological range (10–15 mmHg) [134]. Compared with WT mice, *Cyp1b1*^−/−^ in mice was insufficient to induce murine glaucomatous pathology but increased susceptibility to abnormal IOP-induced retinal axon damage [134].

*(2) Tyr-Deficient Model*. Two mutant genes (*Cyp1b1* and tyrosinase (*Tyr*)) in the mouse model showed that *Tyr* served as a modifier in maintaining the phenotype of the drainage structure and was possibly associated with the severity of PCG [116]. This model showed that *Tyr* deficiency increased the magnitude of the dysgenesis of the structure of the ocular angle, altered the penetrance linked to the inheritance of mutant orthologs of two known human PCG pathogenic genes (*CYP1B1* and *FOXC1*) [116]. Furthermore, oral administration of L-dihydroxyphenylalanine (L-dopa) to pregnant mice improved partial angle dysgenesis of both *Cyp1b1*^−/−^ mice and *Foxc1*^−/−^ mice [116]. Therefore, the pathway involving L-dopa and tyrosinase participates in angle formation or the function of AH drainage structures or functions in the time of prenatal development in mice [116].

*TYR* as a modifier of the phenotype of PCG caused by *Cyp1b1* mutations has not been confirmed in humans [117]. A study in PCG patients found that 16 of 19 nonpermeable families had the same homozygous *CYP1B1* mutations (G61E), resulting in changes in conserved amino acids but did not interrupt protein synthesis as in *Cyp1b1*^−/−^ mice [116, 117]. By analysis of single nucleotide polymorphisms (SNPs), individuals from these nonpenetrant families did not show a significant link to the *TYR* locus [117]. Most of the subjects in this experiment carried recessive missense mutations in *CYP1B1*, and some mutations had been shown to have residual enzymatic activity [117, 135]. Therefore, the limitation of the *Tyr*^−/−^ mouse model is that the role of *TYR* in the eye of humans is different from that of the mouse model, leading to inconsistencies in the role of *Tyr* in *CYP1B1*-induced PCG between mice and humans.

*(3) Angpt1-Deficient or Tek-Deficient Model*. Mouse models harboring a condition by inversion alleles (*Tek*^COIN^ mice) have been developed [17]. In transgenic mice (LoF angiopoietin-*Tek* signaling), PCG-like phenotypes were observed [17]. In *Tek* hemizygous and conditional knockout (KO) mice, a 25% elevation in IOP was found in haploinsufficient mice (*Tek*^+/−^), and IOP levels were moderately related to the degree of SC hypomorphism [17]. *Tek*^+/−^ mice exhibited severe convolutions and focal narrowing of the SC and hypoplastic TM [17]. This evidence supports that the dose of the *TEK* gene plays a significant role in the proper development of AH outflow, and loss of TE*K* signaling underlies PCG with variable expressivity.

Subsequently, mice with *Angpt1*-KO in embryonic day 16.5 (WBΔE16.5) exhibited a severely hypoplastic SC with discontinuous gaps, isolated canal segments, and other glaucoma-like phenotypes [66]. *Angpt1*;*2* double-KO (*Angpt1*;*2*^WBΔE16.5^) mice exhibited a complete absence of SC, while *Angpt2*^WBΔE16.5^ mice showed a normal morphological SC [66]. Compared with WT subjects, *Angpt1*^WBΔE16.5^ mice and *Angpt1*;*2*^WBΔE16.5^ mice exhibited varying degrees of increase in IOP (23.53 ± 1.50 and 35.58 ± 2.01 mmHg, respectively), while *Angpt2*^WBΔE16.5^ mice showed normal physiological IOP, was is ∼15.13 ± 0.41 mmHg [66]. Beyond that, *Angpt1-*KO mice showed that RGC counts decreased, remaining ∼22.38 ± 0.30 cells/10 *μ*m^2^ RGCs [66]. They developed a mouse model with *Angpt1*^p.R494^^*∗*^ mutant allele to investigate variant protein in more detail. *Angpt1*^p.R494^^*∗*^^/ΔE16.5^ mice were shown that *Angpt1*^p.R494^^*∗*^^/ΔE16.5^ mice had a hypomorphic SC, which was similar to the phenotype in *Angpt1*^WBΔE16.5^ mice, and the mutant allele *Angpt1*^p.R494^^*∗*^ was functionally null [66]. Those data demonstrated that *Angpt1* mutation in the *ANGPT/TEK* signaling axis is one of the PCG pathogenic mechanisms.

Mice with PCG may provide evidence that abnormal development in anterior chamber structures and glaucomatous neuro-changes play a key role in revealing the genetic mechanism for PCG. However, abnormal IOP was not reported in some *Cyp1b1*^−/−^ or *Tyr*^−/−^ mouse models. However, compared with humans, the LC is not present, and blood supply for the ONH region is not abundant (absence of choroid and pial vessels), and the number of RGC is less in mice [109]. Thereby, the mouse model is not suitable for the study of specific structures and functions [136]. Moreover, most mouse models are single-gene models, which pose certain limitations regarding the study of multigene interaction diseases in PCG [118].

#### 4.2.2. Zebrafish Model

Ortholog genes are shared among the zebrafish, humans, and mice, more than 70% of human genes have at least one ortholog in zebrafish, and 47% of orthologous genes show a one-to-one relationship in humans and zebrafish [137]. The biologically conserved nature of anterior segment development between zebrafish and humans provides an opportunity for studying inherited eye diseases in recent years [26]. In addition to maintaining significant evolutionary proximity to humans, zebrafish possess unique advantages such as high fecundity, rapid extrauterine development, and transparency during organogenesis, making them ideal for high-throughput drug screening, mechanistic studies, and behavioral genetics [138]. The available genetic manipulation techniques, such as Crispr/Cas9, easily generate zebrafish models with mutation of target genes [139].

*(1) Cyp1b1-Deficient Model*. Cyp1b1 LoF has been studied in zebrafish mainly through morpholino (MO)-mediated knockdown [28, 140]. By this approach, protein expression is inhibited in the early stages of development, and the development of tissues derived from NC cells is affected, further exploring the function of cyp1b1 in early embryonic development [49].

Williams et al. reported that overexpression of *cyp1b1* inhibited ocular fissure closure and caused craniofacial and ocular defects through the RA-independent pathway [28]. Importantly, administration of human *CYP1B1* mRNA to embryos resulted in large colobomas in 71.8 ± 17.9% of embryos and disrupted NC-derived tissue formation, supporting evolutionary conservation of *cyp1b1* function between zebrafish and humans [28]. Additionally, at 4 hours postfertilization (hpf), the volume of embryos from *cyp1b1*-KO zebrafish was 60% of that of the WT group [50]. However, at 168 hpf, no significant histological differences in glaucoma-related structures were observed between *cyp1b1*-KO zebrafish and the WT group [50]. Similar to previous zebrafish models, *cyp1b1*-KO zebrafish exhibited variable craniofacial defects, which may be caused by dysregulation of ECM gene expression induced by *cyp1b1* disruption [50]. The limitation of *cyp1b1*-KO zebrafish is that they do not show a PCG-like phenotype but provide information that tyrosinase does not modify the effect of *cyp1b1* on eye development [28, 50]. It further illustrates that the relationship between *TYR* and *CYP1B1* may vary from species to species.

*(2) Foxc1-Deficient Model*. *Foxc1a* and *foxc1*b of zebrafish are orthologs of human *FOXC1* and *FOXC2*, respectively; the null mutation of *foxc1* in zebrafish models with PCG was generated by Crispr/Cas9 [141]. In zebrafish, *foxc1a* was expressed primarily in NC cells and POM, and *foxc1b* was partially overlappingly expressed with *foxc1*a in the POM [141]. Compared with WT embryos, at 5 days postfertilization (dpf), *foxc1b*^*−/−*^ alone neither affected the cells counts in the RGC layer nor the thickness of the optic nerve nor inhibited *foxc1a* on its own [141]. Then, after administration of *foxc1a*-MO to *foxc1*^*−/−*^ embryos, the number of cells in the RGC layer reduced significantly (146.1 *±* 3.6/section vs. WT 163.5 *±* 3.2/section) [141]. Similarly, in *foxc1b*^*−/−*^ zebrafish with inhibited *foxc1a*, it showed that the thickness of the optic nerve was less than that of the WT group (9.9 *±* 0.55 vs. WT12.2 *±* 0.42 μm) [141]. Moreover, the loss of *foxc1* homologs in zebrafish negatively reduced the expression of atoh7. After the loss of *atoh*7, *pou4f2* expression was reduced, which determined RGC fate [64, 141]. However, loss of *foxc1* homologs did not reduce the level of the marker of amacrine cell fate *scrt1a* [141]. Zebrafish with *foxc1* LoF provides valuable information associated with endophenotypes of glaucoma, suggesting the role of RGC differentiation defects in PCG.

*(3) Guca1c-Deficient Model*. The*guca1c*-KO zebrafish model was developed through Crispr/Cas9 genome editing to study the role of *GUCA1C* LoF in congenital glaucoma and retinal physiology [19]. *Guca1c*-KO zebrafish did not show significant gross external macroscopic changes and PCG-like phenotypes [19]. The thickness of the corneal epithelium in *guca1c*-KO zebrafish was less than that in WT zebrafish (5.1 *±* 0.2 μm vs. 8.6 *±* 0.2 μm) [19]. GCAP3-immunopositive signals were observed in the nonpigmented epithelial cells of the ciliary zone in both *guca1c*-KO and WT zebrafish [19]. In addition, GCAP3 immunoreactivity was not shown in the nonpigmented epithelium, keratocytes, photoreceptors, inner and outer plexiform layers, and the RGC layer in *guca1c*-KO zebrafish [19]. Glial fibrillary acidic protein was upregulated, and RGC apoptosis was observed in Müller cells of *guca1c*-KO zebrafish [19]. The mechanism of *guca1c* LoF may be through nonsense-mediated decay, leading to mRNA degradation and causing the LoF gene product, consequently disrupting the structures of SC and TM through a cGMP-dependent pathway [19, 142]. However, predictive data and functional evidence insufficiently support a gene-disease relationship; further studies are required to demonstrate the pathogenicity of *guca1c* variants in PCG [40].

*(4) Gpatch3-Deficient Model*. In the *gpatch3*^*−/−*^ zebrafish model, *Gpatch3* was expressed in the corneal endothelium and POM-like cells, head cartilages, and skeletal muscles [18]. *Gpatch3*^*−/−*^ zebrafish exhibited variable degrees of abnormality in ocular structures and dose-dependent phenotypes [18]. At 24 hpf, 53% of *gpatch3*^*−/−*^ embryos showed a proportion of lethal phenotypes [18]. At 96 hpf, *gpatch3*^*−/−*^ zebrafish exhibited defects in ocular development relevant to glaucoma in the anterior angle and periocular tissue [18]. Dorsal angle alterations showed decreased silver and foamy cells corresponding to iridophores and xanthophores, respectively [18]. Also, undifferentiated mesenchymal-like cells were accumulated in the anterior segment angle, and hypoplastic development of the ventral angle was observed in the *gpatch3*^*−/−*^ zebrafish model [18]. Other features include increased periocular space and dysplasia of the pharynx and cartilage, indicating a defective ocular structure [18]. Some features resembled that observed in *pitx2-KO* or *foxc1*-KO embryos, which indicated a functional relationship between *gpatch3* and *pitx2* and *foxc1* and provided evidence that *gpatch3* was implicated in PCG [18].

However, because of the different components of the cytoskeleton and different proteoglycans in the TM between zebrafish and humans, zebrafish are not suitable for studying the ultrastructure changes of human PCG [143]. IOP elevation and loss of RGCs were not reported in zebrafish. Furthermore, the ECM was not found in the TMCs of zebrafish, and the annular ligament of zebrafish differs from the TM of humans [143].

## 5. Conclusions

Animal models with various traits play a pivotal role in exploring the pathophysiology and new treatment methods for PCG patients. Animal models are simple representations of complex physiological changes in humans. Each animal model that simulates human PCG has its strengths and weaknesses. Spontaneous animal models are mainly focused on the study of clinical manifestations, pathological changes, and treatment of PCG, providing valuable information on the external phenotype and treatment. The limitation of spontaneous animals is that no more human causative gene is given, except for *LTBP2*. This requires additional animal models to study the molecular mechanisms of human PCG and gene therapy approaches, as well as new molecular techniques to selectively specifically alter the expression of specific genes in specific tissues. Recently, advances in genome editing have specifically modified the expression of individual genes, which has generated new PCG models with specific gene mutations and may be introduced into therapies in the future. Genetic models (mouse and zebrafish) are studied from the causative gene to the cellular and tissue level, providing information on the pathology of PCG from embryonic development to adulthood.

But there are still some problems to be solved. First, some animal models present atypical clinical manifestations, such as a slight or no elevation of IOP. Second, not all genomes of animals are available. Third, the relevance and “translatability” from PCG animal models to humans is limited. Furthermore, topical ocular drug administration usually causes more systemic exposure. Because of the grooming behavior and the relatively large volume of delivery versus the smaller ocular surface area of rodents, rodents rub the remaining dose of the drug from the eyes and then lick the forelimb or remaining hair [21]. Of course, minimal interference factors, reproducibility, and cost-effectiveness should also be taken into account. Therefore, an excellent animal model with PCG should have the following characteristics: (i) markedly elevated IOP; (ii) similarity to the human eye in terms of anatomical structures and physiological function; (iii) optic nerve degeneration similar to human PCG; (iv) animals have available orthologs of human PCG pathogenic genes; and (v) the results of animal models specifically and credibly reflect the pathological process of human PCG.

Due to the anatomical, physiological, and genetic differences between humans and animals, the role of animal models in PCG is to reproduce one or several specific aspects of the disease to provide valuable information to the researchers. Therefore, ongoing efforts focus on the development of a more optimized animal model to simulate the etiology and effects of the treatment of human PCG. The usage of genome editing and available animal genome resources in mice and zebrafish gives a hint that optionally altering the expression of target genes in a given tissue will be an upcoming opportunity to identify new disease-causing genes and mutational loci of PCG. The future development of animal models may provide fascinating information on understanding pathological and genetic characteristics and developing new treatment strategies for PCG.

## Figures and Tables

**Table 1:** Genes related to PCG.

Gene	Cytogenetic location	Locus	Functions in PCG	Possible mechanism	References
*CYP1B1*	2q21-22	GLC3A	Regulates NC migration and maintains the homeostasis of structure-function of TM	RA-independent pathway	[6, 11, 28–30]
*LTBP2*	14q24.3-31.1	GLC3C	Regulates elastogenesis or anchors to TM matrix components	*TGF-β/BMP* pathway^*∗*^	[8, 15, 31, 32]
*FOXC1*	6p25.3	Undesignated	Forms and maintains the ocular surface	*PITX2/DKK2* cascade^*∗*^	[33–35]
*ANGPT1*	8q23.1	Undesignated	Mediates proangiogenic and vascular stabilization by activating *TEK*	*ANGPT1/TEK* pathway	[36, 37]
*TEK*	9p21.2	Undesignated	Regulates the development of SC and TM	*ANGPT1/TEK* pathway	[17]
*GPATCH3* ^*#*^	1p36.11	Undesignated	Regulates NC migration	Unknown	[18, 38]
*GUCA1C* ^*#*^	3p13.13	Undesignated	Changes the volume of SC and TM cells	Unknown	[19, 39, 40]
*CDT6* ^*#*^	1p36.2-36.1	GLC3B	Extracellular matrix organization and function	Unknown	[7, 12–14]
Unknown	14q24	GLC3D	Unknown	Unknown	[9]

^*#*^: candidate pathogenic genes for PCG; ^*∗*^: implicated in glaucoma but unknown in PCG; PCG: primary congenital glaucoma; TM: trabecular meshwork; SC: Schlemm's canal; NC: neural crest; *CYP1B1*: cytochrome P450 family 1 subfamily B member 1; *LTBP2*: latent transforming growth factor-beta binding protein 2; *FOXC1*: forkhead box C1; *ANGPT1*: angiopoietin 1; *TE*K: TEK receptor tyrosine kinase; *GPATCH3*: G-patch domain containing 3; *GUCA1C*: guanylate cyclase activator 1C; RA: retinoic acid; *DKK2*: dickkopf *WNT* signaling pathway inhibitor 2; *PITX2*: paired like homeodomain 2; *TGF-β*: transforming growth factor-beta; *BMP*: bone morphogenetic protein; *CDT6*: cornea-derived transcript 6.

**Table 2:** The advantages and disadvantages of spontaneous animal models with PCG.

Animal	Outcomes	Advantage	Disadvantage	References
Rabbit	IOP elevated as high as ∼40 mmHg Degeneration of the optic nerve	Suitable for histopathology and biochemical studies Large-sized eyes for exploring surgical treatment	Few spontaneous models No human genes given	[22, 101–104]

Rat	IOP elevation ranged from 29 to 42.5 mmHg ∼92 ± 26 RGCs/mm^2^ remaining after 1.5 years	Gene sequence available The dynamics of AH is similar to that of humans	Few spontaneous models The number of RGCs is less	[23, 105–109]

Cat	Elevated IOP ranged from 30 to 40 mmHg (intermittent spikes to 50 to 70 mmHg) The average RNFL thickness varied 8 to 20 *μ*m Loss of the RGCs Remained ∼30,365 optic nerve axons	Novel, longevity model of PCG The sequence homology of *LTBP2* in cats to the human orthologs is 87% over 100% length Provide linkage data and molecular genetics, clinical phenotype, and pathological characteristics consistent with human PCG	Remove mixed phenotypes	[25, 110–113]

Quail	Mean IOP ranged from 18 to 35 mmHg 11%–55% total number of RGCs loss	Observing specifically degenerative changes based on the ganglion cell property	IOP measurement is difficult Mutant genes not described in detail	[27, 114, 115]

PCG: primary congenital glaucoma; AH: aqueous humor; RNFL: retinal nerve fiber layer; IOP: intraocular pressure; RGCs: retinal ganglion cells; *LTBP2*: latent transforming growth factor-beta binding protein 2.

**Table 3:** Pathogenic genes of human PCG confirmed in the genetic model.

Animal	Gene	Outcomes	Challenges	References
Mouse	*Cyp1b1*	The IOP was the modest elevation, ∼11.45 ± 0.20 mmHg 11.1% ± 3.8 collagen in the trabecular beams remained	IOP elevation and PCG phenotype were controversial	[29, 30, 41]
	*Tyr* ^*∗*^	ND	TYR has not been confirmed in human PCG IOP elevation and RGC loss not reported	[116, 117]
	*Tek/Angpt*	IOP elevated in Angpt1^WBΔE16.5^ mice was 23.53 ± 1.50 mmHg, in Angpt1;2^WBΔE16.5^ mice was 35.58 ± 2.01 mmHg; while in Angpt2^WBΔE16.5^ mice was 15.13 ± 0.41 mmHg 22.38 ± 0.30 cells/10*μ*m^2^ RGCs remained in Angpt^1−/−^ mice	IOP in Angpt2^−/−^ mice was normal	[17, 66]

Zebrafish	*cyp1b1*	ND	IOP elevation and RGC loss not reported	[28]
	*foxc1*	The number of cells in the RGC layer was significantly reduced to 146.1 ± 3.6 cells/section. The thickness of the optic nerve was less, about 9.9 ± 0.55 μ*m*	IOP elevation and RGC loss not reported	[26, 118, 119]
	*guca1c* ^*∗*^	The thickness of corneal epithelium was less, which was 5.1 ± 0.2 *μ*m RGC apoptosis	IOP elevation not reported	[19]
	*gpatch3μ*	53% of *gpatch3*^−/−^ embryos showed ocular development defects Increased periocular space and dysplasia of the pharynx and cartilage	IOP elevation and RGC loss not reported	[18]

^*∗*^: implicated in an animal model but still unknown in human PCG; ND: not determined; PCG: primary congenital glaucoma; IOP: intraocular pressure; TM: trabecular meshwork; NC: neural crest; PC: pericyte; WT: wild type; RGC: retinal ganglion cell; *Cyp1b1*: cytochrome P450 family 1 subfamily B member 1; *Foxc1*: forkhead box C1; *Angpt1*: angiopoietin 1; *Tek*: TEK receptor tyrosine kinase; *Gpatch3*: G-patch domain containing 3; *guca1c*: guanylate cyclase activator 1C; *Tyr*: tyrosinase; *atoh7*: atonal homolog 7; *pou4f2*: class IV POU-homeodomain transcription factor 2.

## Data Availability

The data used to support the findings of this study are included within the article.
